# Mutations in *AtPS1* (*Arabidopsis thaliana Parallel Spindle 1*) Lead to the Production of Diploid Pollen Grains

**DOI:** 10.1371/journal.pgen.1000274

**Published:** 2008-11-28

**Authors:** Isabelle d'Erfurth, Sylvie Jolivet, Nicole Froger, Olivier Catrice, Maria Novatchkova, Mathieu Simon, Eric Jenczewski, Raphaël Mercier

**Affiliations:** 1 French National Institute for Agricultural Research (INRA), UR254, Versailles, France; 2 National Center for Scientific Research (CNRS), UPR2355, Gif sur Yvette, France; 3 Research Institute of Molecular Pathology (IMP), Vienna, Austria; The University of North Carolina at Chapel Hill, United States of America

## Abstract

Polyploidy has had a considerable impact on the evolution of many eukaryotes, especially angiosperms. Indeed, most—if not all—angiosperms have experienced at least one round of polyploidy during the course of their evolution, and many important crop plants are current polyploids. The occurrence of 2n gametes (diplogametes) in diploid populations is widely recognised as the major source of polyploid formation. However, limited information is available on the genetic control of diplogamete production. Here, we describe the isolation and characterisation of the first gene, *AtPS1* (*Arabidopsis thaliana Parallel Spindle 1*), implicated in the formation of a high frequency of diplogametes in plants. *Atps1* mutants produce diploid male spores, diploid pollen grains, and spontaneous triploid plants in the next generation. Female meiosis is not affected in the mutant. We demonstrated that abnormal spindle orientation at male meiosis II leads to diplogamete formation. Most of the parent's heterozygosity is therefore conserved in the *Atps1* diploid gametes, which is a key issue for plant breeding. The AtPS1 protein is conserved throughout the plant kingdom and carries domains suggestive of a regulatory function. The isolation of a gene involved in diplogamete production opens the way for new strategies in plant breeding programmes and progress in evolutionary studies.

## Author Summary

In the life cycle of sexual organisms, meiosis reduces the number of chromosomes from two sets (2n) to one set (n), while fertilization restores the original chromosome number. However, in case of failure of meiosis to reduce the chromosome number, the fecundation involving the obtained 2n gametes can lead to the formation of an organism with more than two sets of chromosomes (polyploid). Polyploidization occurred widely in the course of evolution of eukaryotes, especially of plants. Besides, many crops are current polyploids, and 2n gametes have been useful for their genetic improvement by allowing crosses between 2n and 4n species. 2n gametes formation is known to be under genetic control but none of the genes involved were identified. We have isolated and characterised a gene (*AtPS1*) involved in controlling diploid (2n) gamete formation in *A. thaliana*. In the *Atps1* mutant, the second division of meiosis is disturbed, leading to the gathering of chromosomes that had been separated at the first division. Consequently, *Atps1* mutants produce 2n male gametes and spontaneous triploid plants in the next generation. The isolation of a gene involved in diplogamete production opens the way for new strategies in plant breeding programmes and progress in evolutionary studies.

## Introduction

Polyploidy, the condition of organisms having more than two sets of chromosomes, has had a considerable impact on the evolution of many fungi, invertebrate, and vertebrate lineages and is particularly prominent in plants [1],[2]. It is estimated that 95% of ferns are polyploids [3] and that almost all angiosperms have experienced at least one round of whole genome duplication during the course of their evolution [4]. Many important crop plants are currently polyploids or retain the vestiges of ancient polyploid events [5]–[7]. Even plants with small genomes, such as *Arabidopsis thaliana*, have been affected by polyploidy [8],[9]. However, the mechanisms involved in polyploid formation are still poorly understood. For a long time, polyploids were thought to originate from somatic chromosome doubling[10]. The realisation that gametes with somatic chromosome numbers (2n gametes or diplogametes) widely occur in diploid populations as a result of meiotic failure, led to a change of paradigm[11]; it is now believed that 2n gametes are the major route for polyploidy formation, in particular by leading to the formation of triploids, which then may serve as a bridge/step towards even ploidy levels [2], [12]–[15]. 2n gametes are also instrumental in the genetic improvement of several polyploid crops, where useful genes from diploid relatives are incorporated into cultivated genotypes [16],[17].

Given their importance in evolution and crop improvement, 2n gametes have been the focus of a considerable amount of research [12],[18]. The best documented and described meiotic abnormalities leading to 2n gamete formation include abnormal cytokinesis, the omission of the first or second division and abnormal spindle geometry. Co-orientation of 2^nd^ division spindles (parallel spindles or fused spindles) is perhaps the most common mechanism resulting in 2n spore formation [12],[18], most notably in potato [14], and was first described more than eight decades ago [19],[20].

Environmental factors, notably temperature and chemical agents, were shown to affect the frequency of 2n gametes [12],[13],[21]. However, 2n gamete production is under strong genetic control [13]. The genetic determination of 2n pollen production was studied in several species [12] and usually fits the segregation pattern expected for a major locus in a background of polygenic variation. To date, however, none of the genes contributing to high frequency 2n gametes production were identified and characterised at the molecular level [22],[23]. This lack of information has slowed down our understanding of the origins of diplogametes, and limited the potential of diplogametes in crop breeding programmes.

In this paper we describe the isolation and characterisation of the first gene, called *AtPS1* (*Arabidopsis thaliana Parallel Spindle 1*), implicated in the formation of a high frequency of diplogametes in plants. We show that meiosis in *Atps1* mutants generates diploid male spores, giving rise to viable diploid pollen grains and spontaneous triploid plants in the next generation. Analysis of male meiosis showed that during meiosis II spindles are abnormally orientated, with frequent parallel or fused spindles, leading to the production of two sets of chromosomes instead of four at the end of anaphase II. Genetic analyses of the diploid gametes and epistasis experiments demonstrated that diplogamete formation in *Atps1* results from these defects in spindle organisation.

## Results

### Identification of *AtPS1*

*AtPS1* was identified in a screen for genes potentially involved in meiosis using the Expression Angler tool [24], which selects co-regulated genes, in combination with the AtGenExpress tissue data set [25]. We first chose a subset of known meiotic genes (*AtMER3* [*26*], *AtDMC1* [*27*], *SDS* [*28*], *AtMND1* [*29*],[*30*], *AtHOP2* [*31*], *AtMSH5* [32] and *AtSPO11-1* [33]) for which the expression data appeared to be relevant: when one of these genes was used as the query in the Expression Angler tool (with default parameters [24]), other known meiotic genes appeared among the first hits. We thus selected a list of genes that appeared among the first 60 hits in at least one query, and in the first 100 hits in at least two independent queries, using one of these seven genes as bait. Following an additional manual selection, including elimination of genes with known function or essential character, we ended up with a list of 138 candidate genes. We examined the phenotype of one to three lines carrying an insertion in each of these genes (218 lines in total) [34]–[38]. Thirteen genes were not tested because corresponding mutant lines were not found in the databases. We visually screened ∼50 plants of each line obtained from stock centers [35],[39] for reduced fruit length, without genotyping. The meiotic products of plants with reduced fertility were then examined. In two independent lines carrying an insertion in the AT1G34355 gene, plants were found to have slightly reduced fertility and unbalanced meiotic products. Chromosome spreads revealed that these plants were polyploid, prompting us to analyze these lines further. Functional characterization of this gene led us to name it *AtPS1* (see below). Two other genes with meiotic function were identified in the same screen (R. M., unpublished data).

**Figure 1:**
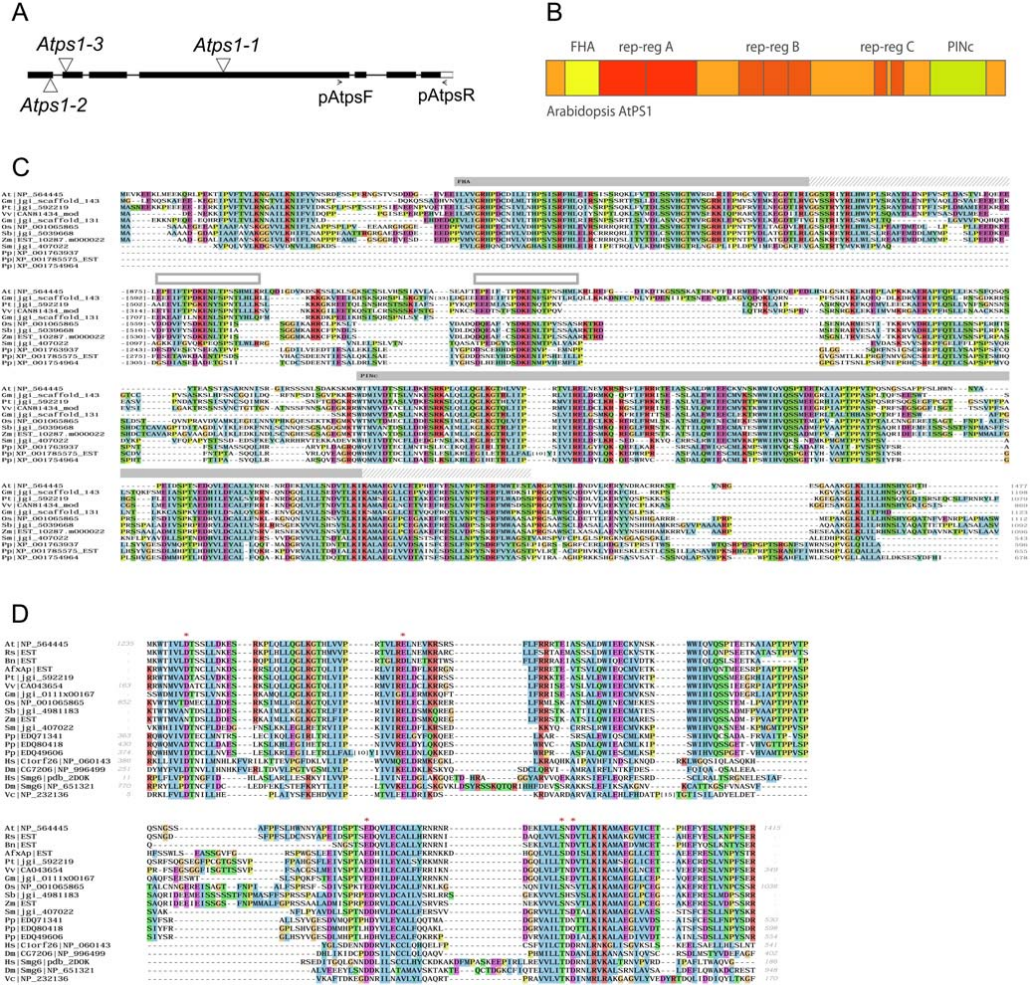
*AtPS1* gene and protein. A) The intron/exon structure of the *AtPS1* gene and location of the three different T-DNA insertions (triangles). The primers used are indicated below the gene diagram. B) Domain architecture of *Arabidopsis thaliana* AtPS1. In addition to the conserved FHA (IPR000253, 64–132aa) and PIN (IPR006596, 1237–1389aa) protein domains, *Arabidopsis* AtPS1 contains three repeat regions (rep-reg) which were further analyzed using HHpredid [67] and REPRO [68]. The CAST program detected a serine bias for the 182–1251 amino acid segment, and a glutamic acid-bias from amino acids 559 to 708 [69]. C) Multiple sequence alignment showing regions of highest sequence conservation among plant AtPS1 proteins. Based on sequence similarity, phylogenetic analysis and domain architecture, the listed plant proteins are likely orthologs of the *Arabidopsis* AtPS1 protein. Full-length plant AtPS1 proteins were aligned and segments of highest conservation were identified using plotcon (EMBOSS package). Non-conserved sequence segments were removed from the alignment. The length of these regions is indicated in box brackets in the corresponding position of the alignment. Domain hits based on a comparison against the Interpro domain database [43] are indicated by gray boxes above the alignment, and the hit-indicators are extended to include adjacent segments which showed sequence and structural conservation. In addition, white boxes highlight a short motif identified as a C repeat element in *Arabidopsis thaliana* AtPS1. Plant AtPS1 proteins typically contain a highly conserved N-terminal FHA domain and C-terminal PINc domain, which are separated by a compositionally biased sequence of variable length. While a single *AtPS1* representative is usually found per species, gene duplication events appear to have occurred in some lineages such as *Physcomitrella patens* and *Glycine max*. The predicted *Physcomitrella* patens co-orthologous sequences do not contain a detectable FHA N-terminal domain, present in all other identified plant AtPS1sequences. This could be due to gain of the FHA domain in the *Tracheophyta* lineage, or due to loss or degeneration of the FHA domain in the *Physcomitrella* lineage. D) Multiple sequence alignment of the plant AtPS1 C-terminal PINc domain and representatives of closely related PINc domain homologs. The closest non-plant AtPS1 PINc domain homologs were identified using profile-based similarity searches such as PSI-BLAST and HHpred [70],[71]. Based on these analyses the AtPS1 PINc domain belongs to a PINc subfamily including human C1orf26, human SMG6, and bacterial PhoH proteins. Conservation of functionally important regions between AtPS1 and human SMG6 PINc domains suggest that the AtPS1 PINc domain might confer nuclease activity/function. Asterisks indicate the residues contributing to the negative charged cavity and potential active site of the human SMG6 PINc domain [46],[72]. Protein sequences are listed using a 2 letter species code followed by a reference to the sequence source. Species codes: At *Arabidopsis thaliana*, Rs *Raphanus sativus*, Bn *Brassica napus*, AfxAp *Aquilegia formosa x Aquilegia pubescens*, Pt *Populus trichocarpa*,Vv *Vitis vinifera*, Gm *Glycine max*, *Os Oryza sativa*, Sb *Sorghum bicolor*, Zm *Zea mays*, Sm *Selaginella moellendorffii*, Pp *Physcomitrella patens*, Hs *Homo sapiens*, Dm *Drosophila melanogaster*, Vc *Vibrio cholerae*. Sequences obtained from the NCBI non-redundant protein database are assigned their NCBI accession number, sequence start/end positions and gene name if available. All other sequences were derived from predicted translations of EST contigs or using preliminary gene predictions made available by the Department of Energy's Joint Genome Institute (JGI).

We amplified the *AtPS1* cDNA (EU839993) by RT-PCR on bud cDNA and sequencing confirmed that it is identical to that predicted in the databases (NM_103158). The *AtPS1* gene contains 7 exons and 6 introns (Figure 1A) and encodes a protein of 1477 amino acids. BLASTp and PSI-Blast [40] analyses showed that the AtPS1 protein is conserved throughout the plant kingdom and contains two highly conserved regions. An FHA domain (forkhead associated domain) was predicted at the N-terminus (CD-search: 65–140 aa, E-value 2e-11) [41], while the C-terminal conserved region shows similarity to a PINc domain as identified using the SMART outlier homologue search (BLAST: PINc, 1237–1389 aa, E-value 1.00e-84) [42], the InterPro superfamily search (SSF88723: PIN domain-like, 1235–1412 aa, E-value 8.8e-09) [43] as well as borderline similarities in CD-search (smart00670: PINc, 1237–1305 aa, E-value 0.21) (Figure 1B and 1C). No close homologs of AtPS1 containing both the FHA and PINc domain were found outside of the plant kingdom. An FHA domain is a phosphopeptide recognition motif implicated in protein-protein interactions and is found in a diverse range of proteins involved in numerous processes including intracellular signal transduction, cell cycle control, transcription, DNA repair and protein degradation [44]. The PINc domain has been predicted to have RNA-binding properties often associated with RNAse activity [45], and this has now been experimentally confirmed [46]. Accordingly, several PINc domain containing proteins are involved in RNAi, RNA maturation, or RNA decay. The highest level of sequence similarity to the AtPS1 PINc domain in eukaryotes was found among others with *S. cerevisiae* Swt1[47], mammalian C1orf26, *Drosophila* CG7206 and SMG6 protein families [45],[48] (Figure 1D).

We investigated the role of the *AtPS1* gene by isolating and characterizing a series of allelic mutants, identified in several public T-DNA insertion line collections [34],[35],[37]. The *Atps1-1* (SALK_078818) and *Atps1-2* (WiscDsLox342F09) insertions are in a Columbia (Col-0) background and are in the fourth exon and first intron, respectively. The *Atps1-3* (FLAG_456A09) insertion is in a Wassilewskija (Ws-4) background and is located in the second exon (Figure 1A). RT-PCR was carried out using the pAtpsF/pAtpsR primers (Figure 1A) on RNA from the *Atps1-3* and *Atps1-1* mutants and no detectable levels of the *AtPS1* transcript were amplified, indicating that these two alleles are null. When the same primers were used on RNA from the *Atps1-2* mutant normal expression levels of this region of the *AtPS1* transcript were observed (data not shown). Nevertheless, the phenotype analysis described below strongly suggests that this third allele is also null.

### Diploid Spores and Pollen Grains in *Atps1*

**Figure 2:**
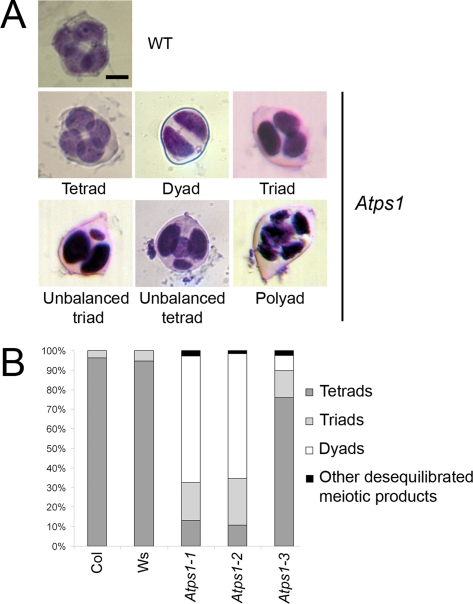
*Atps1* mutants produce dyads of spores instead of tetrads. A) Photos of meiotic products in wild type and *Atps1* mutants. Scale bar = 10 µm. B) Quantification of meiotic products in Ws-4 (n = 92), Col-0 (n = 212), *Atps1-3* (n = 436), *Atps1-1* (n = 1125), *Atps1-2* (n = 554), the *Atps1-1/Atps1-3* F1 (n = 283) and *Atps1-1/Atps1-2* F1 (n = 252).

In *A. thaliana*, male meiosis produces a group of four spores, organised in a tetrahedron, called a tetrad. As expected, male meiotic products in wild type were almost exclusively tetrads (Figure 2). Rarely, (13/304) groups of three spores were also seen but these were most certainly the result of occasional spore superposition. In contrast, the meiotic products in the three independent *Atps1* mutants were characterized by a high frequency of dyads and triads (Figure 2). *Atps1* mutants did not show any other developmental defects. The *Atps1-1* and *Atps1-2* mutants produced a majority of dyads (∼65%). The *Atps1-3* mutant phenotype appeared to be weaker and only 8% of its meiotic products were dyads. Complementation tests between *Atps1-1* and *Atps1-2* and *Atps1-3* and *Atps1-1* showed that these mutations are allelic, and thus demonstrated that the dyads observed in this series of mutants are due to disruption of the *AtPS1* gene.

The *Atps1-3* mutant exhibited a weaker phenotype than the two other alleles, whereas expression analysis suggested that this allele is also null. As this allele was in a different genetic background (Ws-4) to the two others (Col-0), we tested if this difference could be influencing the strength of the phenotype by introducing the Col-0 mutation into the Ws-4 background and *vice versa*. As expected for a background effect, the frequency of dyads increased with successive backcrosses when *Atps1-3* was introduced into Col-0 (from 8% to 58% after four backcrosses) and decreased when *Atps1-1* was introduced into the Ws-4 background (from 64% to 13% after four backcrosses). These results clearly indicate that the frequency of diploid gametes is influenced by multiple genes, with *AtPS1* acting as a major gene.

Pollen grain viability was examined by Alexander staining [49] and showed that in the majority of cases the dyads and triads produced by the mutants result in viable pollen grains (more than 95% in the different *Atps1* mutants : Col: 0 dead pollen grains out of 181 ; *Atps1-1*: 44 dead pollen grains out of 948 ; *Atps1-2*: 3 dead pollen grains out of 363). We did observe however that the pollen grains in mutant plants varied in size (data not shown). We then assessed the ploidy level of *Atps1-1* and *Atps1-2* pollen grains by quantifying spermatic nuclei DNA. Both mutants exhibited two different populations of pollen grains, one corresponding to viable haploid pollen grains (∼40% estimated by maximum likelihood) and another to viable diploid pollen grains (∼60% estimated) (data not shown). These proportions are compatible with the proportion of dyads, triads and tetrads observed in the mutants. In summary, the *Atps1-1* and *Atps1-2* mutants produce a high frequency of viable diploid pollen grains.

### Spontaneous Triploids among *Atps1* Progeny

Next, we measured the ploidy level of the offspring of diploid *Atps1* mutants by flow cytometry. Diploid and triploid plants (30%), but no tetraploid plants, were found among the progenies of *Atps1-1* and *Atps1-2* mutants (*Atps1-1*: 38 triploids out of 130 plants; *Atps1-2*: 30 triploids out of 103 plants). Flow cytometry results were confirmed by karyotyping a subset of 29 plants which were all confirmed to be triploid. This demonstrated that the diploid gametes produced in the *Atps1* mutants are involved in fertilisation and produce viable triploid plants. The appearance of triploids, but not tetraploids, suggests that the *Atps1* mutations only affect male meiosis. As expected for the absence of a female meiotic defect we never isolated triploid plants when ovules from plants with the *Atps1* mutation were fertilised with wild type pollen grains (0 triploids out of 182 plants). When mutant pollen was used for the cross we again observed that 30% of the progeny were triploids (20 triploids out of 56 plants).

The observed frequency of triploid plants (30%) among *Atps1-1* and *Atps1-2* mutant progeny is lower than expected from the frequency of diploid pollen grains produced by these mutants (∼60%). In parallel, more than 50% of seeds obtained by selfing the *Atps1-1* and *Atps1-2* mutants were thinner than wild type, abnormally colored and shaped, and germinated at a rate of 57%, compared to 99.8% in wild type. We do not believe, however, that this seed mortality phenotype infers a possibly essential role for AtPS1 in embryo development, for the following two reasons: 1) 25% (56/210) of the progeny of selfed heterozygotes were mutant and no dead seed was obtained, showing that the *Atps1* mutation does not impair embryo development. 2) The same seed defect (59% of germination) is observed when *Atps1* is crossed as male with wild type as female, which shows that a seed with one functional *AtPS1* allele may show developmental defects. Thus, a likely explanation for the discrepancy between the frequency of diploid pollen grains and triploids in the progeny is abnormal development of triploid seed, which is commonly observed during crosses between plant species with different ploidy levels. These problems are related to the paternal to maternal ratio, which is very important for normal endosperm development [50]. Using C24 and Ler accessions, Scott et al showed that triploid seeds obtained in diploid X tetraploid crosses germinated at a rate of 90%. We obtained stronger germination defects with Col0, suggesting a background effect on the susceptibility to the paternal/maternal ratio. Another, non-exclusive, explanation for the discrepancy could be that haploid pollen grains out-competed diploid pollen grains, which were shown in some cases to germinate more slowly [51],[52]. Nevertheless, approximately 25% of the triploid embryos appear to be able to overcome these constraints.

### The Mechanism Leading to Dyads Production

**Figure 3:**
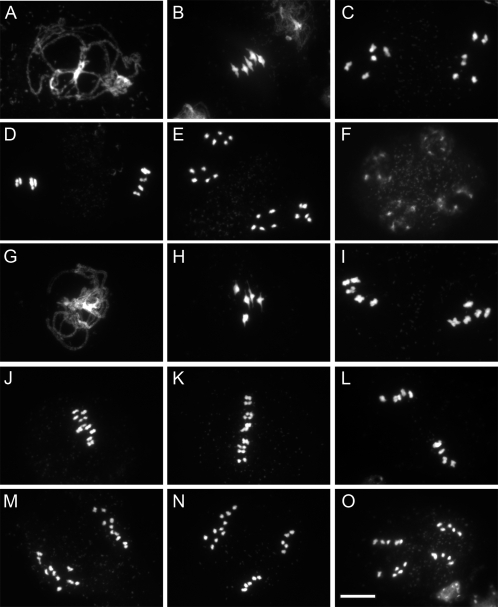
Meiosis I is normal but meiosis II is defective in *Atps1* mutants. A–F) Wild type meiotic chromosome spreads. A) pachytene. B) metaphase I. C) anaphase I. D) metaphase II. E) Anaphase II F) telophase II. G–O *Atps1-1* meiosis. G) pachytene. H) metaphase I. I) anaphase I. J–L) metaphase II. M–O) Anaphase II. M) dyad. N) triad. O) tetrad. Scale bar = 10 µm.

To unravel the mechanisms leading to dyad production in *Atps1-1*, we investigated chromosome behaviour during meiosis (Figure 3). Chromosome spreads showed that the meiosis in the *Atps1-1* mutant progresses normally and is indistinguishable from the wild type until the end of the telophase I. Synapsis was complete, chiasmata formed (the cytological manifestation of crossovers) and bivalents were seen (compare Figure 3G–I with Figure 3 A–C, for example). At metaphase II, however, differences were seen compared to wild type with the 10 chromosomes aligned in a same plane, causing abnormal looking figures, rather than two well separated metaphase II plates containing five chromosomes each (Compare Figure 3J–K with Figure 3D). In rare cases, metaphase II in *Atps1* did appear normal however (Figure 3L). At telophase II, we observed dyads (two sets of 10 chromosomes, Figure 3M), triads (2 sets of five chromosomes and one set of 10, Figure 3N) and normal tetrads (4 sets of 5 chromosomes, Figure 3O). These observations are consistent with the previous finding that *Atps1* meiotic products are a mixture of dyads, triads and tetrads.

**Figure 4:**
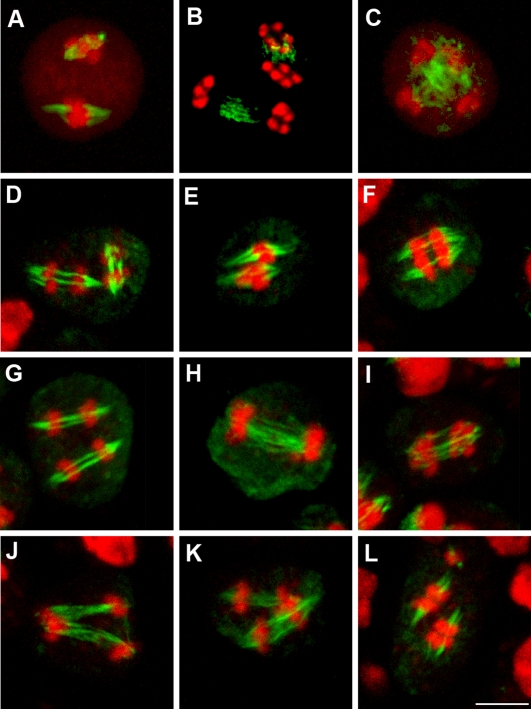
The relative orientation of spindles at metaphase II is disrupted in *Atps1*. A, B, C) Wild type spindles at metaphase II, anaphase II and telophase II, respectively. D–L) *Atps1-1* meiocyte at metaphase II / anaphase II. Chromosomes were stained by propidium iodide or DAPI (red, false color) and microtubules by immunolocalisation (green, false color). Scale bar = 5 µm.

These results and specifically the alignment of the 10 chromosomes at metaphase II suggested that the meiotic spindles in *Atps1* mutants are defective at this stage. We thus examined spindle organisation by immunolocalisation with an alpha-tubulin antibody (Figure 4). In wild type plants the majority of metaphase II spindles were roughly perpendicular to each other (Figure 4A), leading to four well separated poles at anaphase II (Figure 4B) and the formation of tetrads (Figure 4C). In the *Atps1* mutant, while individual metaphase II / anaphase II spindles appeared regular in most cases their respective orientation was aberrant. The majority of cells had parallel spindles (Figure 4D to 4G), fused spindles (Figure 4H and 4I) or tripolar spindles (Figure 4J and 4K). This defect in spindle orientation explains the appearance of triads and dyads. These conformations cause chromatids, that had been separated at meiosis I, to gather at anaphase II. Occasionally, three to four sets of chromosomes encompassed by a spindle were dispersed in the cell at metaphase II (Figure 4L). This type of defect is probably the cause of the few unbalanced meiotic products observed in the *Atps1* mutants.

The name *AtPS1* for *Arabidopsis thaliana Parallel Spindle 1* was chosen due to the high percentage of parallel spindles produced by the corresponding mutants.

**Figure 5:**
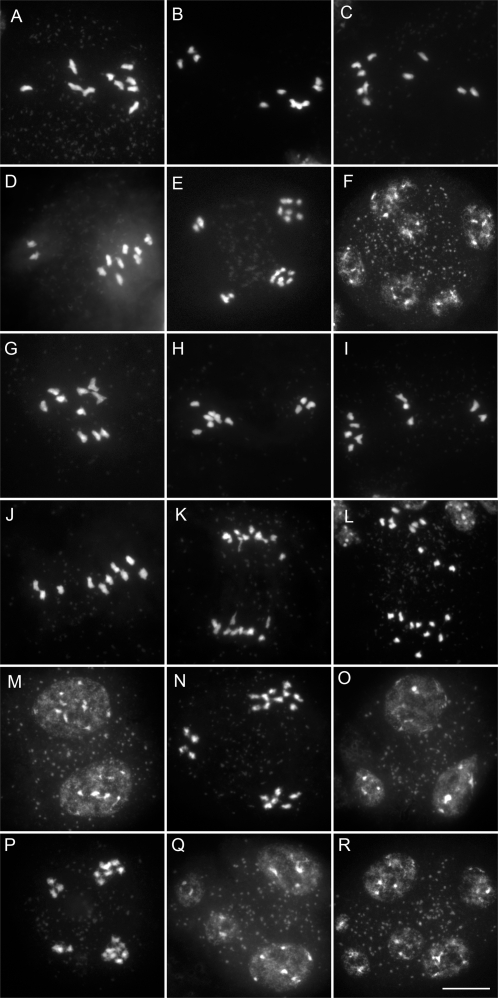
Unbalanced chromosome distribution at meiosis I does not affect dyad formation in *Atps1*. A–F) *Atspo11-1* meiotic chromosome spreads. A) metaphase I. B, C) anaphase I. D) metaphase II. E) Unbalanced Tetrad. F) Polyad. G to R) *Atps1-1/Atspo11-1* meiosis. G) metaphase I. H, I) anaphase I. J) metaphase II. K) Anaphase II. L, M) Balanced dyad II. N. O) Triad. P, Q) Unbalanced tetrad. R) Polyad. Scale bar = 10 µm.

Thus, parallel spindles at metaphase II in the *Atps1* mutants appear to be leading to the formation of dyads. This proposed mechanism implies that unbalanced chromosome segregation at meiosis I would have no impact on the final distribution of chromosomes in the resulting dyad. To test this hypothesis we constructed a double *Atspo11-1/Atps1* mutant. The *Atspo11-1* mutant (N646172, *Atspo11-1-3*) [53] displays an absence of bivalents at meiosis [33] (Figure 5A) leading to frequent unbalanced first divisions (Figure 5B) that can be associated with lagging chromosomes (Figure 5C). At metaphase II, unbalanced plates are seen (Figure 5D), leading to unbalanced tetrads (Figure 5E). Lagging chromosomes at anaphase II, lead to multiple metaphase II plates and then polyads with more than four nuclei (Figure 5F).

In the *Atspo11-1/Atps1* background the first division was identical to the single *Atspo11-1* phenotype. We observed 10 univalents at metaphase I (Figure 5G), leading to missegregation at anaphase I, with two sets of unbalanced chromosomes (Figure 5H) or three sets because of lagging chromosomes (Figure 5I). At metaphase II, we regularly observed two unbalanced metaphase plates, which had a tendency to be parallel instead of perpendicular (Figure 5J). This led to the formation of dyads which were always balanced (Figure 5K to 5L, n = 44). We also observed triads with one set of 10 chromosomes caused by an unbalanced first division followed by the fusion of two of the four second division products (Figure 5N), which is highly consistent with our proposed mechanism. We also observed unbalanced tetrads (Figure 5P and 5Q), expected since the *Atps1* mutation is not fully penetrant, and polyads due to lagging chromosomes at the first division (Figure 5R).

**Figure 6:**
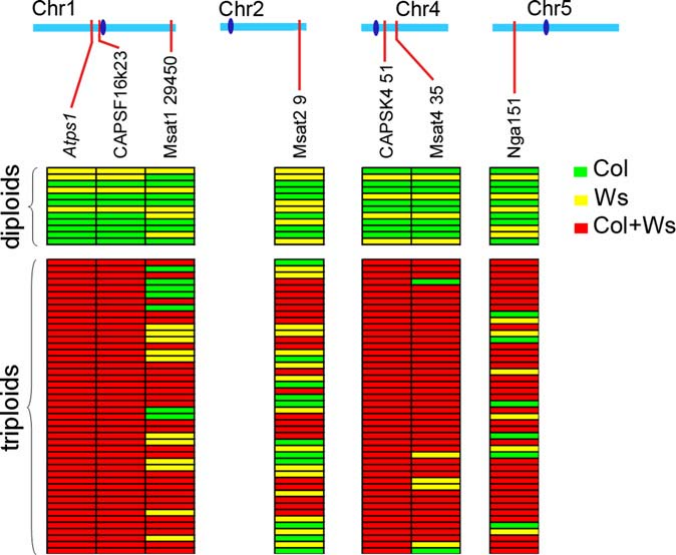
Diploid gametes are heterozygous at centromeres. Diploid and triploid offspring of the *Atps1-1*(Col-0) / *Atps1-3*(Ws-4) ♂×Ler ♀ cross was genotyped for several genetic markers. For each marker plants bearing only the Col-0 allele are in green, plants bearing only the Ws-4 allele are in yellow and plants bearing both the Col-0 and Ws-4 alleles are in red. The Ler alleles are present in all the plants because it was used as the female parent in the cross. The position of each marker (red) and the centromeres (dark blue) are indicated along the chromosomes.

Another prediction of the proposed mechanism is that centromere distribution should resemble that seen during mitosis, e.g., any heterozygosity at the centromeres should be retained in the diploid gametes. Indeed, in *Atps1*, the first division is identical to wild type, with the co-segregation of sister chromatids and separation of homologous chromatids. Thus, in the case of a heterozygous genotype, *A/a*, at the centromere, following the first division the two *A* alleles will end up at one pole, and the two *a* alleles at the opposite pole. In wild type, the second division separates the two sisters leading to four spores with one chromatid. In *Atps1*, the second division would regroup the products of the first division, thus grouping the *a* and *A* allele in each cell, leading to systematic heterozygosis at the centromere. Because of recombination, loci unlinked to centromeres should segregate randomly. We tested this prediction by taking advantage of the two genetic backgrounds of the *Atps1-1* (Col-0) and *Atps1-3* mutants (Ws-4). F1 plants bearing the two mutations – thus mutant for *AtPS1* and heterozygous for any Col-0/Ws-4 polymorphisms – were crossed as male to a third genetic background Landsberg erecta (Ler). Karyotyping and genotyping of the obtained plants for trimorphic molecular markers provided direct information regarding the genetic make up of the pollen grain produced by the mutant (Figure 6). All the diploid pollen grains tested had the predicted genetic characteristics. They were systematically heterozygous at centromeres and segregating–because of recombination–at other loci. These results confirm that the “parallel spindle” defect is indeed the cause of at least the vast majority of 2n pollen in *Atps1*.

## Discussion

In this study, we identified and described the *AtPS1* gene and a corresponding set of mutants that produce pollen grains which are up to 65% diploid and give rise to numerous triploid plants in the next generation. Another *Arabidopsis* mutant that leads to severe meiotic defects and almost sterility [54],[55] was recently described and reported to produce diploid female gametes [56], but at a frequency of several orders of magnitude lower than the frequency of 2n gametes induced by the *Atps1* mutation. By combining cytological and genetic analyses, we carried out a detailed investigation of the mechanism responsible for these 2n pollen grains in *Atps1*, and established that they result from abnormal orientation of spindles at meiosis II. Interestingly, defects in meiosis II spindles are the most common known mechanisms responsible for the formation of 2n spores, [12],[18] and are the main source of the 2n pollen which is extensively used in potato breeding programmes [14]. In potato, a major locus called *ps* was shown to be responsible for the parallel spindle phenotype more than 30 years ago [57], but the corresponding gene is still to be identified. As was observed in different *ps* potato lines, *Atps1* mutations only affect male meiosis and the frequency of dyads formed depends on the genetic background. The *AtPS1* gene is conserved in higher plants (Figure 1C) and is therefore a good candidate for the gene behind the major *ps* locus of potato [14].

The fact that *Atps1* mutations only affect male meiosis points to a difference in regulation between male and female 2n gametes production. This phenomenon was previously described for mutations that had a specific impact on either male or female meiosis [12],[23],[55],[56],[58]. In the case of parallel spindles, it may stem from the 3-dimension organization of the spores (e.g. tetrahedron in male vs linear or multiplanar arrays in female [59]).

The AtPS1 protein has two domains, a FHA (ForkHead Associated) domain, a phosphopeptide recognition domain found in many regulatory proteins and a PINc domain, which is found in proteins involved in RNA processing [48]. In fungi/metazoa, the AtPS1 PINc domain shows highest similarity with the PINc domains of the Swt1/ C1orf26/ CG7206 and SMG6 protein families followed by SMG5, Dis3 and others. The mammalian C1orf26 and Drosophila CG7206 genes encode related proteins of unknown function, but Interestingly both are overexpressed in testis and ovaries, which is consistent with a putative meiotic role [60],[61]. SMG6 is an essential component of the Nonsense Mediated RNA Decay (NMD) machinery that degrades mRNAs containing premature translation termination codons. SMG6 also plays a role in RNAi [45],[48]. The SMG6 PINc domain has RNA degradation activity [46]. These features suggest that *AtPS1* plays a regulatory function, perhaps *via* RNA decay, which may directly control the orientation of metaphase plates/spindles or be related to meiotic cell cycle control. There is growing evidence that NMD and its components have important functions in various cellular processes, including the cell-cycle [62]. A link between RNA decay and the control of meiosis progression was recently suggested because SMG7, which is a NMD essential component, is involved in progression through meiotic anaphase II in *Arabidopsis* [63].

Further studies involving *AtPS1* should shed light on the poorly understood process of meiosis II. The isolation of a gene involved in 2n gamete production has important implications for deciphering meiosis mechanisms, as well as potentially fundamental applications in evolution studies and plant breeding programmes.

## Materials and Methods

### Growth Conditions

*Arabidopsis* plants were cultivated as described in [64]. For germination assays and cytometry experiments *Arabidopsis* were cultivated *in vitro* on *Arabidopsis* medium [65] at 21°C with a 16h day/8h night photoperiod and 70% hygrometry.

### Genetic Analysis

The *Atps1-1* (SALK_078818) and *Atps1-2* (WiscDsLox342F09) lines were obtained from the European Arabidopsis stock centre [39]. The *Atps1-3* (FLAG_456A09) insertion is from the Versailles T-DNA collection[35]. Plants were genotyped by PCR (30 cycles of 30 s at 94°C, 30 s at 56°C and 1 min at 72°C) using two primer pairs. For each line the first pair designated is specific to the wild type allele and the second pair is specific to the T-DNA insertion.

*Atps1-3*: EQM96L (5′ACATCTCCCTTGTCGTAAC3′) and EQM96U (5′ATCTCTCAATCGTTCGTTC3′); EQM96L and tag3 (5′ CTGATACCAGACGTTGCCCGCATAA3′). *Atps1-1*: N578818U2 (5′TCGGAGTCACGAAGACTATG3′) and N578818L (5′CAGTCTCACTGATTATTCCTG3′); N578818U2 and LbSalk2 (5′GCTTTCTTCCCTTCCTTTCTC3′). *Atps1-2*: N851945U (5′AAGGCTGATATTCTGATTCAT3′) and N851945L (5′CTCTTGTTGGTCCGTATCTTA3′); N851945U and P745 (5′AACGTCCGCAATGTGTTATTAAGTTGTC3′). *spo11-1-3*: N646172U (5′AATCGGTGAGTCAGGTTTCAG3′) and N646172L (5′CCATGGATGAAAGCGATTTAG 3′); N646172L/ LbSalk2.

Genetic markers used to genotype *Atps1-1/Atps1-3*×Ler F1 triploid and diploid plants (40 cycles of 20 s at 94°C, 20 s at Tm and 30 s at 72°C): Microsatellite msat1.29450 (located on chromosome I at position 29450001) was amplified (Tm = 57°C) using 5′TCCTTTCATCTTAATATGC3′ and 5′TCTGTCCACGAATTATTTA3′ primers. Microsatellite Msat4.35 (Tm = 58°C) (located on chromosome 4 at position 7549125) was amplified using 5′CCCATGTCTCCGATGA3′ and 5′GGCGTTTAATTTGCATTCT3′ primers. Microsatellite NGA151 (Tm = 58°C) (located on chromosome 5 at position 4669932) was amplified using 5′GTTTTGGGAAGTTTTGCTGG3′ and 5′CAGTCTAAAAGCGAGAGTATGATG3′ primers. The 2 primer pairs specific for the *Atps1-1* and *Atps1-3* TDNA borders were used as a centromeric marker of the chromosome 1. CAPS markers Seqf16k23 (physical position: 14481813) and CAPSK4 51 (physical position: 5078201) were used as centromeric markers for chromosome 1 and 4, respectively. CAPS Seqf16k23 was amplified (Tm = 60°C) using 5′GAGGATACCTCTTGCTGATTC3′ and 5′CCTGGCCTTAGGAACTTACTC3′ primers and observed after TaqI digestion. CAPS CAPSK4 51 was amplified (Tm = 60°C) using 5′CAATTTGTTACCAGTTTTGCAG3′ and 5′TGAGTTTGGTTTTTTGTTATTAGC3′ primers and observed after MnlI digestion.

### Cytology and Flow Cytometry

Final meiotic products were observed as describe in [28] and viewed with a conventional light microscope with a 40× dry objective. Chromosomes spreads and observations were carried out using the technique described in [33]. The DNA fluorescence of spermatic pollen nuclei was quantified using open LAB 4.0.4 software. For each nucleus the surrounding background was calculated and subtracted from the global fluorescence of the nucleus. Meiotic spindles were observed according to the protocol described in [55] except that the DNA was counter-stained with DAPI. Observations were made using an SP2 Leica confocal microscope. Images were acquired with a 63× water objective in xyz and 3D reconstructions were made using Leica software. Projections are shown. Cells were imaged at excitation 488 nm and 405 nm with AlexaFluor488 and DAPI respectively. *Arabidopsis* genome sizes were measured as described in [66] using tomato Lycopersicon esculentum cv “Montfavet” as the standard. (2C = 1.99 pg, %GC = 40.0%).

### RT-PCR

Arabidopsis total RNA was extracted using the QUIAGEN RNA kit.

Reverse transcription was done on 5 µg of total RNA using oligo(dT)_18_ as primer. The RevertAid M-MuLV Reverse Transcriptase enzyme (Fermentas) was used according to the manufacturer's instruction. RT-PCR was carried out on 1 µl of cDNA using the pAtpsF and pAtpsR primers and the following PCR conditions: 30 cycles of 30 s at 94°C, 30 s at 56°C and 1 min at 72°C.
